# Immunohistochemical and Molecular Characteristics with Prognostic Significance in Diffuse Large B-Cell Lymphoma

**DOI:** 10.1371/journal.pone.0098169

**Published:** 2014-06-02

**Authors:** Carmen Bellas, Diego García, Yolanda Vicente, Linah Kilany, Victor Abraira, Belen Navarro, Mariano Provencio, Paloma Martín

**Affiliations:** 1 Laboratory of Molecular Pathology, Instituto de Investigación Sanitaria, Hospital Universitario Puerta de Hierro-Majadahonda, Madrid, Spain; 2 Unidad de Bioestadística Clínica, Hospital Universitario Ramón y Cajal, CIBER Epidemiología y Salud Pública (CIBERESP), Madrid, Spain; 3 Department of Hematology, Hospital Universitario Puerta de Hierro-Majadahonda, Madrid, Spain; 4 Medical Oncology Service, Onco-hematology Research Unit, Instituto de Investigación Sanitaria, Hospital Universitario Puerta de Hierro-Majadahonda, Madrid, Spain; Health Canada and University of Ottawa, Canada

## Abstract

Diffuse large B-cell lymphoma (DLBCL) is an aggressive non-Hodgkin lymphoma with marked biologic heterogeneity. We analyzed 100 cases of DLBCL to evaluate the prognostic value of immunohistochemical markers derived from the gene expression profiling-defined cell origin signature, including MYC, BCL2, BCL6, and FOXP1 protein expression. We also investigated genetic alterations in *BCL2*, *BCL6, MYC* and *FOXP1* using fluorescence in situ hybridization and assessed their prognostic significance. *BCL6* rearrangements were detected in 29% of cases, and *BCL6* gene alteration (rearrangement and/or amplification) was associated with the non-germinal center B subtype (non-GCB). *BCL2* translocation was associated with the GCB phenotype, and BCL2 protein expression was associated with the translocation and/or amplification of 18q21. *MYC* rearrangements were detected in 15% of cases, and MYC protein expression was observed in 29% of cases. FOXP1 expression, mainly of the non-GCB subtype, was demonstrated in 37% of cases. Co-expression of the MYC and BCL2 proteins, with non-GCB subtype predominance, was observed in 21% of cases. We detected an association between high FOXP1 expression and a high proliferation rate as well as a significant positive correlation between MYC overexpression and FOXP1 overexpression. MYC, BCL2 and FOXP1 expression were significant predictors of overall survival. The co-expression of MYC and BCL2 confers a poorer clinical outcome than MYC or BCL2 expression alone, whereas cases negative for both markers had the best outcomes. Our study confirms that DLBCL, characterized by the co-expression of MYC and BCL2 proteins, has a poor prognosis and establishes a significant positive correlation with MYC and FOXP1 over-expression in this entity.

## Introduction

Diffuse large B-cell lymphoma (DLBCL) is the most common B-cell lymphoma and is curable in more than 60% of patients treated with rituximab plus cyclophosphamide, doxorubicin, vincristine and prednisone (a treatment known as R-CHOP) [1]. DLBCL is an aggressive non-Hodgkin lymphoma with marked biologic heterogeneity. This heterogeneity has been recognized in clinical presentation and morphology as well as in molecular and cytogenetic features. As a result, numerous studies have attempted to assess the prognostic value of individual biomarkers [2]. Traditionally, DLBCL has been classified by the morphology and immunophenotype of the malignant B-cells; however, more recent reports describe molecular classifications for DLBCL.

Cell-of-origin classification based on gene expression profiling (GEP), has demonstrated that a germinal center (GC) profile predicts better survival than an activated B-cell-like (ABC) profile among DLBCL patients treated using chemotherapy with or without rituximab [3]–[6]. GEP is not readily applicable in routine clinical practice; therefore, several immunohistochemical algorithms that combine immunostaining of CD10, BCL6 and MUM1/IRF4 have been developed for use on paraffin-embedded tissues [7]–[9]. The correlation between the gene expression profiling subgroups of DLBCL and those defined as GC and ABC using immunohistochemistry is highly variable, and the prognostic value of these algorithms has not been validated [10]–[12].

In addition to CD10, BCL6 and MUM1/IRF4, the expression of other proteins encoded by genes such as *LM02* or *FOXP1*, which belong to the GC and ABC signature, respectively, can now be determined using immunohistochemical analysis of routinely processed tissues [13]–[15]. FOXP1 is a transcription factor involved in cell signaling and regulating gene expression; the protein is essential for early B-cell development. Upregulation of FOXP1 mRNA expression has been reported in response to normal B-cell activation. FOXP1 protein expression has been detected in 40-60% of DLBCL cases, and strong expression of FOXP1 has been associated with poorer prognosis in patients with some B-cell lymphomas [16], [17]. It has also been reported that MALT lymphomas showing strong FOXP1 expression are at risk for transformation into an aggressive form of DLBCL [18].

Several cytogenetic translocations have been found in DLBCL with the most common involving the *BCL2, BCL6* and *MYC* loci, though the prognostic relevance of these translocations is controversial. *BCL2* gene rearrangement is associated with GC DLBCL and has been identified as an adverse prognostic factor in this DLBCL subtype [19], [20]. However, the prognostic significance of a *BCL2* breakpoint was not determined in other studies [21]–[25]. *BCL6* translocations are observed with a higher frequency in the non-GC DLBCL subtype, although studies on the influence of *BCL6* gene rearrangement in patient outcomes have yielded conflicting results [21], [24], [26]–[29].

*MYC* translocation has been reported to occur in DLBCL with a frequency of 5%-10%. Because such cases have unfavorable prognoses [24], *MYC* status has become a critical factor in selecting patients for more intensive regimens. Recently, a novel monoclonal antibody that targets the *N*-terminus of the MYC protein was shown to provide sensitive and specific staining of nuclear MYC in paraffin-embedded tissue [30], [31]. This antibody was able to identify cases of DLBCL with a *MYC* translocation, raising the question of whether patients with MYC IHC-positive DLBCL should be considered for more aggressive therapy.

In this study, we used tissue microarray (TMA) analysis to evaluate the prognostic value of immunohistochemical markers derived from the GEP-defined cell origin signature, including MYC and FOXP1 protein expression. We also investigated FOXP1 amplification and the status of *BCL2*, *BCL6* and *MYC* breakpoints using fluorescence in situ hybridization (FISH) and assessed their prognostic significance.

## Material and Methods

### Case selection and tissue microarray construction

#### Ethics Statement

Written informed consent was obtained for all patients and clinical investigation has been conducted according to the principles expressed in the Declaration of Helsinki. The study was approved by the Research Ethics Board of our hospital (Comité Ético de Investigación Clínica del Hospital Universitario Puerta de Hierro-Majadahonda).

Biopsies from 100 DLBCL patients diagnosed between 1996 and 2011 in the Pathology Department of Puerta de Hierro Hospital were enrolled in this study. The group comprised 85 cases of primary (de novo) DLBCL and 15 secondary (recurrent or transformed) lymphomas.

A tissue arrayer (Beecher Instruments, Silver Spring, MD, USA) was used to construct the TMAs. Tumor specimens were reviewed by two investigators (CB and PM), with the DLBCL diagnosis based on the 2008 WHO classification criteria [1]. Paraffin sections were examined to select involved areas. Representative tumor regions were identified and marked in the paraffin blocks. In each case, two cylinders 1-mm in diameters were selected from two different areas, along with 11 different controls to ensure the quality, reproducibility and homogeneous staining of the slides. These controls were provided by four B and T lymphoma-derived cell lines (Raji, HUT 78, Toledo, and Karpas 422), four normal lymphoid tissue (two tonsils and two reactive lymph nodes), two Hodgkins lymphoma samples and one Burkitt lymphoma. Cases not evaluable on the TMAs were studied as whole-tissue sections.

### Immunohistochemistry

Tissue microarray blocks were sectioned at a thickness of 3 µm and dried for 16 h at 56°C before being dewaxed in xylene, rehydrated through a graded ethanol series and subsequently washed with phosphate-buffered saline. Antigen retrieval was achieved by heat treatment in a pressure cooker for 2 min in 10 mM citrate buffer (pH 6.5). Endogenous peroxidase was blocked prior to staining the sections. Immunohistochemical staining was performed on the sections using routine staining protocols and the following antibodies: BCL2, clone 124, 1∶50 dilution (Dako, Glostrup, Denmark); BCL2, clone E17, 1∶100 dilution (Epitomics, Burlingame, CA, USA); BCL6, clone PG-B6p, 1∶25 dilution (Dako); MYC, clone Y69, 1∶50 dilution (Epitomics, Burlingame, CA, USA); FOXP1, rabbit polyclonal, 1∶200 dilution (Abcam ab16645, Cambridge, UK); MUM-1, clone MUM1p, 1∶25 dilution (Dako) and CD10, clone 56C6, 1∶50 dilution (Novocastra, Newcastle upon Tyne, UK). Ki-67 immunohistochemical staining analyses were performed manually using the MIB-1 clone, 1∶50 dilution (Dako). The percentage of positive tumor nuclei was manually given a score between 0 and 100%. A cut-off value of greater than or equal to 80% was used to define a high proliferation index in accordance with the recommendations of the Southwest Oncology Group [32]. Cases in which ≥50% of tumor nuclei showed staining were considered MYC-positive, according to criteria proposed by Kluk *et al.* [31] on the basis of their finding that cases with *MYC* translocation have >50% of tumor nuclei positive for MYC protein. Consistent with other reported studies and the findings of Johnson *et al* [33], BCL2-positivity was defined as ≥50% cells showing cytoplasmic staining. High, uniform expression of FOXP1, with a cut-off of 80% as proposed by Barrans *et al.* [16] was used to determine FOXP1 positivity.

### Fluorescence *in situ* hybridization

Interphase FISH analysis was performed on 3-µm TMA tissue sections using commercial dual-color break-apart probes (Abbott Molecular, Des Plaines, IL, USA), according to previously described methods [34], [35]. Samples were analyzed using a Leica DM 5000B fluorescence microscope. Probes for *MYC*/8q24, *BCL2*/18q21 and *BCL6*/3q27 were used for detecting translocations. The signals from 200 intact, non-overlapping nuclei were analyzed, and the hybridization signal scoring was performed according to the protocol described by Ventura *et al.* [36]; the cut-off to consider a case rearranged was established with the mean +3 SD of split nuclei in the reference samples. This threshold was 10% for the three break-apart probes. Samples with three or more fusion signals in ≥10% of cells were considered positive for amplification. Slides were analyzed independently by two scorers (YV and PM) using a 100x oil-immersion objective.

Detection of *FOXP1* amplification was performed using the SureFISH 3p13 probe to label *FOXP1* together with the centromeric SureFISH Chr3 CEP (Agilent Technologies, Cedar Creek, TX, USA). According to the criteria described by Hoeller *et al.* [17] high- level amplification was defined as presence of >10 gene signals and *FOXP1* gains were defined as the presence of tumor cell nuclei with three or more signals exceeding the mean 3 SD [37] of false positive signals from 100 nuclei in the reference controls, i.e., presence of additional *FOXP1* gene signals in >11% of evaluated tumor cell nuclei.

### Statistical analysis

Statistical analysis was performed using SPSS 15.0 for Windows (SPSS Inc., Chicago, IL, USA). Categorical variables were analyzed using the chi square test and, if necessary, Fisher's exact test. Two-tailed *p* values of less than 0.05 were considered statistically significant.

Survival curves were calculated according to the Kaplan-Meier method, and differences between curves were evaluated using the log-rank test. Overall survival (OS) was calculated from the date of diagnosis to the date of death or last follow-up. Patients who died during treatment or as a consequence of treatment were considered to be deaths due to DLBCL. Patients known to have died from causes unrelated to DLBCL or its treatment were omitted from the survival analysis. Progression-free survival (PFS) was measured from the date of pathologic diagnosis until lymphoma progression or death as a result of any cause.

## Results

### Patient characteristics

The clinical features of the patients are summarized in Table 1. There were 53 males and 47 females with a median age of 61 years (range 18–88 years). Fifty patients had nodal DLBCL, and 50 samples were obtained from extranodal sites. Involved extranodal sites included the stomach, breast, testis, small intestine, lung, mediastinum, liver, central nervous system, skin and thyroid. A total of 91 patients received immunochemotherapy including adriamycin-containing regimens (86 received R-CHOP, and 5 patients received R-ESHAP). Three patients received no treatment, and no treatment data were available for six patients.

**Table 1:** Clinicopathological characteristics of diffuse large B-cell lymphoma.

Patients (*n = 100*)	
Median patient age (range), years	61 (18–88)
Gender (*n*)	
Male	53
Female	47
Presentation	
Nodal	50
Extranodal	50
Phenotype (Han's)	
Germinal center	51
Non-Germinal center	49
IPI risk group	
Low risk (0–2)	62
High risk (3–4)	38
***Immunohistochemistry***	
BCL2 protein expression	62
BCL6 protein expression	67
MYC protein expression	29
MYC and BCL2 co-expression	21
FOXP1 protein expression	37
Ki67≥80%	31
***FISH***	
*BCL2* rearrangement only	12
*BCL2* amplification	27
*BCL2* rearrangement and amplification	8
*BCL2* normal	53
*BCL6* rearrangement only	22
*BCL6* amplification	23
*BCL6* rearrangement and amplification	7
*BCL6* normal	48
*MYC* rearrangement only	7
*MYC* amplification	15
*MYC* rearrangement and amplification	8
*MYC* normal	70
*FOXP1* amplification	3

### Immunohistochemical and FISH studies

Following the immunohistochemistry (IHC) algorithm described by Hans *et al*., 51 patients (51%) were diagnosed with the GCB subtype, and 49 patients (49%) with non-GCB DLBCL.

*BCL2* translocations were detected in 20 of 100 DLBCL patients (20%); 8 patients showed both translocation and amplification, and 12 showed translocation without amplification. Fifty-three cases (53%) demonstrated a normal pattern, and 47 cases (47%) exhibited *BCL2* gene alterations (translocation and/or amplification). Amplification of *BCL2* was detected in 35 of 100 cases. BCL2 protein expression (clone E17) was positive in 62 cases, whereas 38 cases were BCL2-negative. Using clone 124, we detected 77 BCL2-positive cases and 23 BCL2-negative cases. BCL2 protein expression was associated (*p* = 0.004) with translocation and/or amplification of 18q21, and *BCL2* translocation was associated with the GCB phenotype (*p = *0.003). No correlation was observed between BCL2 expression and cell origin phenotype.

Forty-eight cases (48%) demonstrated a normal *BCL6* pattern, and 52 cases (52%) had showed *BCL6* gene alterations. *BCL6* translocations were detected in 29 of 100 DLBCL cases (29%); seven exhibited both abnormalities (translocation and amplification), and 22 had translocation without amplification. Amplification of *BCL6* was detected in 30 of 100 samples. BCL6 protein expression was detected in 67 cases, and 33 samples were BCL6-negative. No association was found between *BCL6* translocation and BCL6 protein expression (*p* = 0.8), or between *BCL6* translocation and cell-of-origin phenotype (*p = *0.09) or extranodal disease (*p* = 0.5). *BCL6* gene alteration (translocation and/or amplification) was associated (*p* = 0.05) with the non-germinal center B subtype (non-GCB).

**Figure 1:**
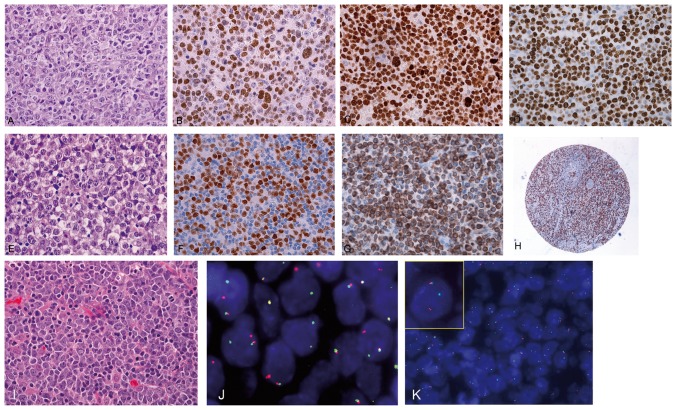
DLBCL case showing centroblastic morphology in hematoxylin and eosin (A). The phenotypic profile shows extensive nuclear positivity for MYC (B), FOXP1 (C) and KI-67, with high proliferation fraction (D). Immunoblastic variant of DLBCL (E–H). The majority of cells show prominent central nucleoli in hematoxylin and eosin (E). The phenotypic profile shows extensive positivity for MYC (F) and BCL2 (G) staining. (H) Low magnification of tissue microarray core illustrating the MYC expression. Double-Hit BCL6/MYC DLBCL (I–K). Proliferation of intermediate monotonous population of intermediate-sized to large-sized lymphoid cells (I). FISH for BCL6 (J) and MYC (K) shows translocation of these genes with 1 normal fusion signal and a split signal.

*MYC* genetic alterations were detected in 30 cases (30%) (Table 1). Of 100 DLBCL cases, 15 cases (15%) were positive for *MYC* translocation and 85 cases showed no *MYC* rearrangement. Seven of 15 cases demonstrated only rearrangement and eight showed both rearrangement and amplification. Another 15 cases presented extra *MYC* signals without gene translocation (Table 2). Six of those 15 cases (40%) had *MYC* rearrangement as the sole translocation, with neither *BCL2* nor *BCL6* translocation. Simultaneous rearrangements of *MYC* and either *BCL2* or *BCL6* (*MYC* double hits) were identified in 7 cases, of which 4 had *MYC* and *BCL2* rearrangements and 3 had *MYC* and *BCL6* rearrangements (Figures 1J, 1K). Two additional cases presented triple hit *MYC-BCL2-BCL6* rearrangements. No correlation was found between *MYC* rearrangement and any Han's category.

**Table 2:** Correlation between FISH status and MYC immunohistochemistry results.

FISH MYC (8q24)	IHC MYC Negative	IHC MYC Positive	Total
Without Alterations	57	13	70
Rearranged	1	6	7
Amplified	12	3	15
Rearranged and amplified	1	7	8
Total	71	29	100

MYC staining was interpretable in all cases. Twenty-nine tumors showed MYC expression in ≥50% of cells (Figures 1B, 1F, 1H), and 71 cases were negative. Because the cut-off for MYC immunohistochemistry used in most studies is 40% [33], we performed the same analyses using this cut-off value with no change in results.

A significant positive correlation was observed between *MYC* gene alteration (amplification and/or translocation) and nuclear MYC expression (*p*<0.0001), with 69 cases negative for both assays (8q24 translocation and IHC) and 13 cases positive for FISH translocation and IHC (Table 3). The remaining cases comprised 16 samples without translocation (including three cases that showed an amplification signal) and with MYC nuclear expression, and two cases demonstrating translocation without protein expression (Table 2). Thirteen cases were positive for protein expression without translocation or gene amplification. A trend toward significance (*p* = 0.07) was noted between *MYC* amplification and MYC protein expression. Overexpression of MYC protein was found in 29 tumors, of which 45% (13 cases) may be explained by the presence of translocation. Both MYC protein expression and *MYC* rearrangement were associated with a high proliferation rate (Ki-67≥80%) (*p* = 0.004 and *p* = 0.04, respectively).

**Table 3:** Correlation between MYC immunohistochemistry and *MYC* rearrangement.

FISH MYC (8q24)	IHC MYC Negative (n = 71)	IHC MYC Positive (n = 29)
MYC Rearranged	2	13
MYC not rearranged	69	16

*p*-value <0.00010.

Twenty-one DLBCL samples (21%) co-expressed MYC and BCL2 proteins (Figures 1F, 1G) with non-GC subtype predominance (14 non-GC and seven GC subtype).

Uniformly high expression of FOXP1 was found in 37 cases and was negative in the other 63 samples. As expected, FOXP1 expression was more frequent in the non-GC DLBCL subtype and correlated with a high proliferation rate (*p*<0.001) (Figures 1C, 1D). Fifteen cases (15%) showed a *FOXP1* locus gain, 12 of which had trisomy of chromosome 3 and three of which had isolated *FOXP1* gain. FOXP1 protein expression was independent of *FOXP1* amplification (*p* = 0.28). High-level amplifications were not detected in our study.

A significant positive correlation (*p* = 0.001) has been found between MYC overexpression and FOXP1 overexpression. Within the MYC IHC positive group, 18 cases were FOXP1 positive (18 of 29; 62%) and 11 cases were FOXP1 negative (11 of 29; 38%). In the MYC negative group, only 19 cases (26%) were FOXP1 positive, whereas 52 cases were negative.

Chromosomal alterations affecting *BCL2, BCL6* and *MYC* are common in DLBCL. In the present study, 74% of cases showed alteration of at least one gene, with *BCL6* representing the most frequently translocated gene, followed by *BCL2* and *MYC* (29%, 20% and 15%, respectively). The most frequent gene involved in amplification was *BCL2,* followed by *BCL6* and *MYC* (35%, 30% and 23%, respectively).

Triple rearrangements were detected in two cases; one a GC-cell subtype in the gastric mucosa, and the other a nodal non-GC B-cell lymphoma. Both cases had unfavorable outcomes with disease progression and death within 1 year. Concurrent *BCL2* and *MYC* rearrangements were found in four cases comprising two extranodal (gastric and brain) and two nodal lymphomas. Three of these patients were non-responders, and one patient presented with early relapse.

Rearrangement of *BCL6* and *MYC* was detected in three patients, all of whom had nodal lymphomas with non-GC phenotypes. All patients showed no response to chemotherapy and disease progression with short OS (median <6 months).

Two cases showed double rearrangement simultaneously involving the *BCL2* and *BCL6* genes. One patient was a 73-year-old man diagnosed 4 years prior with a grade 1/2 follicular lymphoma that was not treated. A punch biopsy of the scalp revealed DLBCL with a GC phenotype. He was treated using R-CHOP but did not respond and died shortly thereafter. The other patient was a 31-year-old woman with a leg-type lymphoma that was refractory to multi-agent chemotherapy. She developed central nervous system involvement and had a poor outcome.

There was a higher incidence of double hit in relapsed lymphomas (27%) than in primary lymphomas (8%). Similarly, we found a higher incidence of positive MYC and BCL2 protein expression in relapsed DLBCL than in primary cases (33% and 19% respectively).

### Factors associated with clinical outcome

**Figure 2:**
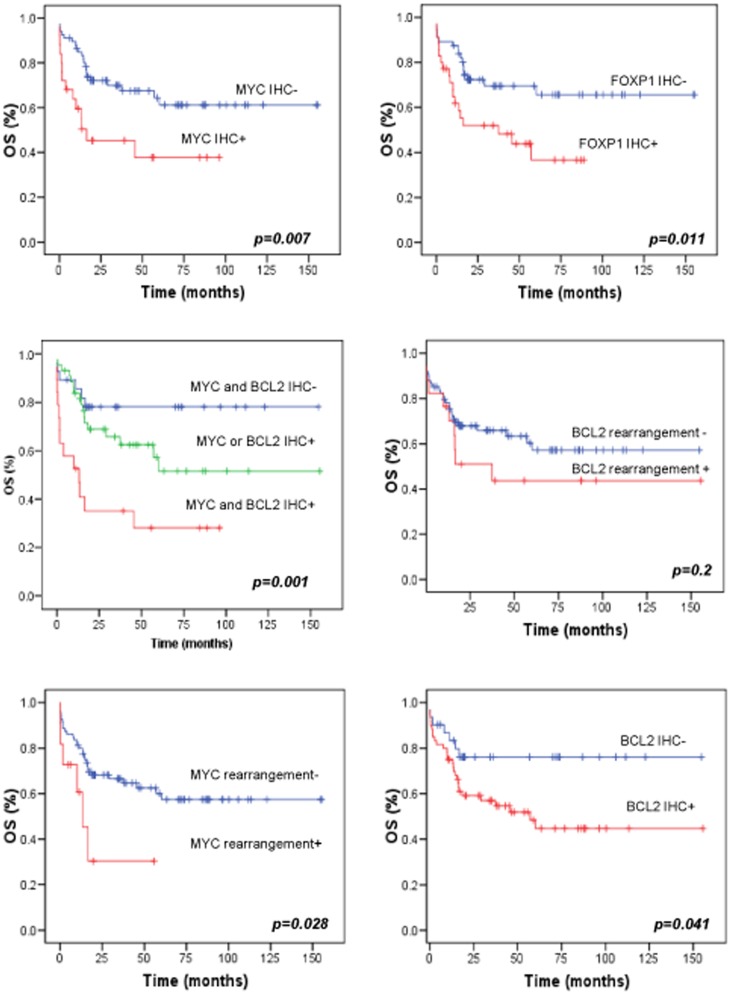
Overall survival of patients with DLBCL based on alterations in MYC, BCL2 and FOXP1. Kaplan Meier curves represents OS according to A) MYC protein expression, B) FOXP1 protein expression, C) presence of MYC and BCL2 expression, D) *MYC* translocation, E) *BCL2* translocation, F) BCL2 protein expression.

We analyzed prognostic factors in 91 patients for whom we had adequate clinical follow-up. Sixty of the 91 patients (66%) with assessable responses to therapy reached a complete remission. The follow-up time was 38.5 months (range, 1–155 months). Thirty-five patients died of their disease. Univariate analysis of the prognostic factors revealed that IPI>2, MYC expression, BCL2 expression and FOXP1 expression (using an IHC method) were significant predictors of OS (*p* = 0.002, *p* = 0.007, *p* = 0.041 and *p* = 0.011, respectively) (Table 4, Figure 2). The presence of *MYC* gene translocation constituted a significant risk factor based on univariate analysis (EFS: *p* = 0.012; OS: *p* = 0.028). No differences in terms of OS or EFS were found between cases with *MYC* amplification and those without *MYC* amplification (*p* = 0.8). The five-year OS according to MYC protein expression was 70% for negative cases versus 40% for positive cases.

**Table 4:** Univariate analysis of the biologic factors predictive of survival in DLBCL.

	Overall survival		
	Hazard ratio	95%CI	*P*
MYC immunohistochemistry	2.45	1.24–4.81	0.007
*MYC* rearrangement	2.62	1.07–6.41	0.028
BCL2 immunohistochemistry	2.31	1.18–6.16	0.041
*BCL2* rearrangement	1.53	0.72–3.27	0.259
BCL6 immunohistochemistry	0.56	0.23–1.10	0.080
FOXP1 immunohistochemistry	2.29	1.18–4.42	0.011
Ki67≥80%	1.69	0.85–3.36	0.129
MYC and BCL2 co-expression	2.24	1.38–3.62	0.001
IPI >2	3.01	1.12–12.6	0.002

Univariate analysis indicated that *BCL2* rearrangement was not significantly associated with OS (*p* = 0.25). BCL6 positive protein expression, but not FISH rearrangement, demonstrated a trend towards better OS than negative BCL6 cases (*p* = 0.08). Multivariate analysis using the Cox model which included MYC rearrangement, MYC expression, BCL2 expression, FOXP1 and IPI, indicated that elevated IPI score (HR = 2.25; 95%CI = 1.52–10.12;*p* = 0.020), and MYC expression (HR = 2.18; 95%CI = 1.10–4.31;*p* = 0.024) were independently associated with patient outcomes.

Consistent with the results of recent studies [30], [33], [38], [39], co-expression of MYC and BCL2 confers a worse outcome (Figure 2C) than isolated MYC or BCL2 expression (*p* = 0.001).

The cohort included cases of both primary and relapsed DLBCL; we therefore performed survival analysis for each group. The relapsed DLBCL group showed a five year OS rate of 36% *versus* 59% in primary DLBCL, but the number of patients in the relapsed group was too small (15 cases) for the analysis to be statistically significant.

## Discussion

DLBCL represents a clinically and genetically heterogeneous group of tumors. Various morphologic, immunohistochemical, cytogenetic and molecular subgroups have been identified. Gene expression profiling studies have provided prognostically relevant information and indicate a major division determined by whether the DLBCL cell of origin is a germinal center B-cell (GCB)-like disease or an activated B-cell (ABC)-like disease. However, molecular classification is not feasible in routine clinical practice, and the predictive value of the immunohistochemically defined GC and non-GC phenotypes remains controversial. In this series, the GC/non-GC phenotype made according to Hans' algorithm had no prognostic impact, which is consistent with the majority of studies of R-CHOP patients [15], [40], [41].

Although no single genetic aberration typifies DLCBL, recurrent chromosomal translocations involving the *BCL6*, *BCL2* and/or *MYC* genes occur in approximately 50% of DLBCL cases [38].

In our series, *BCL6* rearrangements were detected in 29% of DLBCL cases, a result consistent with those reported by other groups [23], [38], [39]. *BCL6* gene alteration (rearrangement and/or amplification) was significantly correlated with cell-of-origin: 62% of samples with *BCL6* translocation were of a non-GCB subtype, as compared with 44% of samples without *BCL6* gene translocation. According to other authors, no association has been observed between *BCL6* rearrangement and either protein expression or extranodal localization [26], [40]. Consistent to other reports [27], we did not detect any influence of *BCL6* rearrangement on prognosis in DLBCL patients. In contrast, BCL6 protein expression conferred a trend towards better outcomes (*p* = 0.08).

BCL2 overexpression is observed in approximately 60% of DLBCLs. Of these, only 15–20% showed translocation (14;18), indicating that other mechanisms also regulate the protein's expression [1], [19], [21], [41], [42]. In our series, BCL2 protein expression was significantly associated with translocation and/or amplification of 18q21 (*p* = 0.004). Consistent with Visco *et al.* [43], BCL2 overexpression had prognostic value only with respect to the GCB subtype, and not the ABC DLBCL subtype. No correlation has been found between BCL2 expression (using dual antibody techniques) and the cell-of-origin phenotype.

*MYC* translocation is a defining feature of Burkitt lymphoma but is not specific, as it may also occur in other B-cell lymphomas. A small subgroup of DLBCL patients show genetic translocations involving *MYC*; these patients have poor prognosis [33], [44], [45].

In our series, *MYC* amplification was more frequent than translocation. Extra *MYC* signals were detected in 23 samples, 15 of which lacked any *MYC* rearrangement. *MYC* rearrangements were detected in 15 cases (15%), and MYC protein expression was detected in 29 of 100 (29%) cases. A good correlation was found between MYC protein expression and translocation (p<0.0001). These results are consistent with results reported by other studies using the same antibody. Thirteen cases showed MYC protein expression without translocation or gene amplification, suggesting that MYC expression in DLBCL is controlled not only by genetic events but also by other signaling pathways.

MYC and BCL2 protein co-expression has been described as an important and robust tool to risk-stratify patients with DLBCL [33]. Patients with simultaneous expression which creates a synergistic effect that promotes proliferation (*MYC*) and blocks apoptosis (*BCL2*) have worse outcomes. Twenty-one DLBCL samples co-expressed the MYC and BCL2 proteins and were predominantly of the non-GC subtype. This high frequency of MYC-BCL2 co-expression in the non-GC subtype may contribute to the overall poor prognosis of patients in this subset [30], [46]. This present study supports immunohistochemical analysis as a robust and reproducible tool for identifying high-risk DLBCL patients using the thresholds recommended in the current guidelines.

The deregulation of FOXP1 expression plays an important role in lymphoma development, although the underlying molecular mechanism is poorly understood. FOXP1 is targeted by chromosome translocations in MALT lymphoma and DLBCL [47], [48], wherein high-level protein expression is associated with a poor prognosis.

FOXP1 mRNA overexpression is a prognostic indicator for poorer outcomes in DLBCL patients treated using CHOP or R-CHOP [49]. On the protein level, the role of FOXP1 expression has been controversial [13], [16]. In our series, 37% of cases showed FOXP1 expression, which was predominantly associated with the non-GC DLBCL subtype [50]. In results consistent with those of Hoeller *et al.* [17] our study demonstrates that assessing FOXP1 protein expression was of greater prognostic importance with respect to OS (*p* = 0.011) than assessing whether DLBCL was of GCB or non-GCB origin. The incidence of *FOXP1* gene amplification in DLBCL is low [37]. Here, we used FISH to determine *FOXP1* copy number changes and found only three cases of gene amplification without trisomy of chromosome 3. There were no clinical or histological features associated with the amplification. Protein expression was independent of *FOXP1* gene amplification; however, other mechanisms may be implicated in FOXP1 deregulation in DLBCL.

Interestingly, a significant association has been reported between the high FOXP1 expression and high proliferation rates. Here, we also found a significant positive correlation (*p* = 0.001) between MYC overexpression and FOXP1 overexpression. Within the MYC IHC positive group, 62% of cases were FOXP1 positive. In the MYC negative group, only 26% of cases were FOXP1 positive. Craig *et al.* [51] described FOXP1-positive cases that also expressed MYC in a series of gastric DLBCL and suggested the existence of a novel MYC- and FOXP1-dependent pathway mediated by microRNAs. miR-34a has tumor-suppressive properties in DLBCL, and its expression is directly regulated by MYC. “MYC expression is negatively regulated by miR-34a, implying that the loss of miR-34a expression in DLBCL may further de-repress MYC and perpetuate the oncogenic consequences of MYC dysregulation” [51]. In addition, miR-34a is implicated in B-cell development and targets FOXP1 by binding to ≥1 of 2 predicted seed regions in the *FOXP13*'-UTR [52]. Therefore, aberrant expression of MYC causes the repression of miR-34a and, consequently, FOXP1 deregulation.

Double-hit (DH) and triple-hit (TH) lymphomas are transformed B-cell lymphomas that are generally defined by the presence of *MYC* gene rearrangement together with either *BCL2* and/or *BCL6* gene rearrangement. In our series, 10 cases (10%) showed two or three translocated genes (TH/DH lymphomas). Three cases had *MYC* rearrangements and extra *BCL2* signals, and according to the findings of Li *et al.* [53], the outcomes in such cases have been very poor.

Our data, although limited by a small sample size, indicate that DH and TH lymphomas are aggressive and have poor treatment response rates.

Cases showing DH and co-expression of MYC and BCL2 were more frequent in relapsed DLBCL than in primary cases. This incidence is consistent with data reported by Pedersen *et al.* [54] although in that study all relapsed cases had previous follicular lymphoma and the GCB immunophenotype. In our series none of the DH cases had previous follicular lymphoma and the distribution between GCB and non GCB categories was even.

Given the heterogeneous nature of the DLBCL, analysis of *MYC*, *BCL2* and *BCL-6* alterations, at both the gene and protein levels, may provide important prognostic information to help identify a high-risk group of patients who do not respond well to current treatment regimens and are most likely to benefit from novel therapeutic approaches.

During the review process of this report a new GEP assay for cell of origin assignment in paraffin-embedded tissue has been pre-published with prognostic implications [55]. However, these findings will require confirmation in an additional series to clarify if this new assay (cell of origin assignment in formalin-fixed paraffin-embedded tissue) is more effective than MYC and BCL2 analysis for identifying a subgroup of DLBCL patients with poor outcomes.
